# Building effective engagement for implementation with i-PARIHS: a collaborative enquiry into paediatric pain care in the emergency department

**DOI:** 10.1186/s12913-022-07740-w

**Published:** 2022-03-12

**Authors:** Suzanne Williams, Samantha Keogh, David Herd, Sharonn Riggall, Roselyn Glass, Clint Douglas

**Affiliations:** 1 School of Nursing and Centre for Healthcare Transformation, Faculty of Health, Queensland University of Technology (QUT), QLD Brisbane, Australia; 2 Acute Pain Service, Children’s Health Queensland Hospital and Health Service, Queensland Health, Brisbane, QLD Australia; 3 Alliance for Vascular Access Teaching and Research Group (AVATAR), Menzies Health Institute Queensland, Griffith University, Brisbane, QLD Australia; 4 Emergency Department, Children’s Health Queensland Hospital and Health Service, Queensland Health, Brisbane, QLD Australia; 5 Metro North Hospital and Health Service, Brisbane, QLD Australia

**Keywords:** Context, Culture, Emergency department, Facilitation, Implementation, i-PARIHS, Paediatric, Pain management

## Abstract

**Background:**

Pain is a central and distressing experience for children in the emergency department (ED). Despite the harmful effects of pain, ED care often falls short of providing timely and effective pain relief. Knowledge translation research targeting systems of care holds potential to transform paediatric pain care. This article reports on the first stages of an implementation project aimed at embedding effective and sustainable practice change in an Australian children’s hospital ED.

**Methods:**

The integrated Promoting Action on Research Implementation in Health Services (i-PARIHS) framework underpinned a cooperative process of engagement to establish a practitioner-led, interprofessional research collaborative. The Kids Pain Collaborative (KPC) aimed to co-design innovation in paediatric ED pain care, facilitating an extensive reconnaissance of research evidence, clinician and family experiences, and local evaluation data. This critical appraisal of the context and culture of pain management generated foci for innovation and facilitation of implementation action cycles.

**Results:**

Engaging in a complex process of facilitated critical reflection, the KPC unpacked deeply embedded assumptions and organisational practices for pain care that worked against what they wanted to achieve as a team. A culture of rules-based pain management and command and control leadership produced self-defeating practices and ultimately breakdowns in pain care. By raising a critical awareness of context, and building consensus on the evidence for change, the KPC has established a whole of ED shared vision for prioritising pain care.

**Conclusions:**

In-depth key stakeholder collaboration and appraisal of context is the first step in innovation of practice change. The KPC provided a space for collaborative enquiry where ED clinicians and researchers could develop context-specific innovation and implementation strategy. We provide an example of the prospective application of i-PARIHS in transforming ED pain care, using a collaborative and participatory approach that has successfully enabled high levels of departmental engagement, motivation and ownership of KPC implementation as the facilitation journey unfolds.

**Supplementary Information:**

The online version contains supplementary material available at 10.1186/s12913-022-07740-w.

## Introduction

Children in the emergency department (ED) often wait in pain. Despite decades of research, timely and effective pain care at the bedside is hindered by the failure to translate evidence-based knowledge into practice at a systems level. In the short term, acute pain increases a child’s anxiety, fear and distress, also heightening the pain experience [1–3]. Longer term effects include greater risk of healthcare avoidance and chronic pain conditions [1, 3]. To prevent the harmful consequences of pain, a recent Lancet Commission on paediatric pain care proposed four simple but transformative goals—make pain matter, understood, visible and better—advocating knowledge translation as a central strategy to drive urgent service improvements [3]. Greater engagement of frontline clinicians to optimise pain care in the ED with local innovation and practitioner-led workforce development is at the heart of this transformation agenda [4].

In our recent integrative review of organisational interventions for paediatric pain care in the ED, we argued for more collaborative and theoretically-driven approaches to implementation [5]. Much of the existing research has ignored the crucial importance of working with context, deep engagement with staff around what is often contested evidence for pain management practice and placing family involvement at the heart of change and evaluation strategies [5]. Based on these recommendations our approach has been to adopt an implementation framework—the integrated Promoting Action on Research Implementation in Health Services (i-PARIHS)—to guide the complex process of engagement with clinicians and families, appraisal of context and culture of pain management in the ED setting, and analysis of evidence to build consensus on the focus of practice change [6]. This article reports on the findings and implications of these first stages as part of the larger implementation project.

### Working with i-PARIHS

The i-PARIHS framework has evolved over more than 20 years of practice development and implementation research, building on an extensive synthesis of empirical, theoretical and experiential literature [6–9]. As a framework that supports complex bottom up change, Harvey and Kitson [6] argue the construct of “evidence-based innovation” best captures the way (new) knowledge is generated and negotiated as it finds its way into clinical practice. While research evidence is given prominence, a multifaceted view of evidence recognises clinical experience, patient experience and local evaluation data are all key sources of evidence for building consensus around the need for change and shaping the proposed innovation [6]. Whatever the starting point for implementation—a discrete intervention or clinical guideline with a well-established evidence base, or simply a focus for practice improvement with a participatory process of emergent innovation—i-PARIHS begins with a consensus-building process, assessing the compatibility and fit of available evidence with local context, practice and clinician values.

This article reports on the prospective application of i-PARIHS to plan and execute a research project aimed at facilitating innovation and knowledge translation in paediatric pain care. We have collaborated with ED clinicians and families to analyse the context and assess the contextual readiness for innovation in pain care, mapping our findings on to the i-PARIHS framework to explore implications for facilitating and embedding sustainable practice change.

### Aims

- To establish an interprofessional and authentic clinical-academic research collaborative to optimise pain care in a tertiary paediatric ED.
- To explore and build consensus around the different dimensions of evidence for paediatric pain care in the ED
- To appraise the context and culture of pain care in the ED to inform an integrated facilitation model for implementation.
- To evaluate the prospective application of the i-PARIHS framework in guiding research design, facilitation and implementation approaches.

## Methods

### Study design

Working with the i-PARIHS framework we focus here on two essential phases, “clarify and engage” and “assess and measure”, to develop a facilitation strategy tailored to the ED context, recipients and innovation [6]. During the first phase, we scoped the project, consulting with and engaging key stakeholders from the inner context. Developing these relationships enabled us to establish the Kids Pain Collaborative (KPC), an interprofessional clinical-academic research collaborative focused on the common goal of optimising pain care in a tertiary paediatric ED. Throughout the second phase, the KPC conducted an extensive appraisal of evidence for innovation, recipients and context to assess readiness for practice change [6]. These first two phases of the larger implementation project were completed over 21 months from March 2019 to October 2020. The first 12 months enabled engagement with the ED team and joint development of the research protocol and ethics application. Contextual appraisal took 9 months, with in-kind funding for ED and University staff time. The KPC then sought and obtained external grant funding to support the next phases of implementation and evaluation.

The project used a blended internal-external model of facilitation. External facilitators included two nursing professors and a paediatric emergency nurse practitioner (NP) who led the project as an embedded researcher in the ED [10]. The NP also has a clinical-academic appointment with the facility and so was able to engage stakeholders across the hospital, building the credibility of the project. The external team was complemented by committed internal facilitators who were experienced ED clinicians with comprehensive knowledge of the context and pain management processes.

### Setting

The ED is set in a large metropolitan teaching paediatric hospital which provides specialist paediatric clinical services to Queensland and northern New South Wales in Australia. It has a large nursing, medical and administration team caring for more than 74,000 children per year, from birth to 16 years. Clinical services and tertiary level education programs are provided by a permanent team of more than 200 nurses and 40 senior medical officers (SMOs), supported by allied health, ancillary, security and administration staff. As a major paediatric teaching hospital, physicians in training, nursing and medical students are also routinely rotated through the ED for varying lengths of time, ranging from 6 weeks to 6 months.

### Participants

To ensure assessment of the context was multi-dimensional all recipients were engaged: clinicians and families [6]. As this project was facilitated across the whole of ED, all clinicians participated. To be inclusive of recipients external to ED we extended a hospital-wide invitation to join the KPC, establishing a core group of 12 ED nurses (including three NPs), a SMO, three nurse leaders from the hospital Acute Pain Service and three experienced nurse researchers (academic and health-service based). As the KPC became established, three ED nursing members were appointed as internal facilitators and we recruited six local pain champions (ED nurses) to raise awareness and advocate for optimal pain management. Three SMOs acted informally as pain champions supporting internal facilitators.

We invited 11 parents, including seven mothers and four fathers, to participate in separate individual interviews to explore family experiences of ED pain care. These participants were purposively selected by KPC members to capture a range of ages, painful presentations, and experiences. We completed a retrospective pain audit of all children presenting from birth to 16 years during the year before the project commenced (see Supplementary file for sample characteristics).

### Methods of data generation and collection

Analysis and integration of data across the first two phases of the project contributed to a comprehensive understanding of evidence and context to shape our facilitation plan. First, the external facilitators engaged with key stakeholders including formal ED nursing and medical leaders to scope and establish the project [11]. The primary investigator recorded these meetings using a reflective journal. Subsequently, the KPC facilitated a comprehensive reconnaissance of pain management evidence relevant to the ED context, meeting at two-week intervals over 9 months. Allowing sufficient time for collaborative enquiry was critical to the success of our appraisal—enabling the KPC to become embedded in the ED and fully exploring all relevant sources of pain management evidence as group reflection and learning evolved. Facilitation of KPC meetings enabled curious questions and established a safe, participatory process where critical questioning was normalised. It supported the KPC to explore the context and culture of pain care and tailor facilitation strategies to fit [6]. Consent was obtained from all members of the KPC to attend meetings and audio record the discussions which were transcribed by the primary investigator (PI).

#### Research evidence

Our integrative literature review directly addressed the project aims and was presented for discussion at several KPC meetings [5]. We reviewed and circulated other local, national and international sources of scientific evidence [1, 12–14]. Clinicians tended to refer to local policy as their most trusted source of evidence rather than peer-reviewed literature. We also established which evidence-based paediatric pain management tools were already available to ED clinicians.

#### Family experience

Family experience was captured through interviews and transcriptions shared with the KPC for discussion and reflection. Initially conducted face-to-face, the majority of interviews were undertaken online when the COVID-19 pandemic precluded meeting in person. Families of children presenting with a range of painful conditions were selected and approached by KPC clinicians before leaving the ED. A two-stage consent process was used whereby families were provided with verbal and written information about the study and consented to being contacted by the PI. At follow-up the family member was asked again for verbal consent to proceed with the interview and for it to be recorded. We used emotional touchpoints to explore family experiences [15]. Emotional touchpoints representing key points in pain care over the ED journey were identified (e.g., pain assessment at triage) and used to explore their pain experience. A range of emotional words were presented to families during the interview to assist them in expressing how they felt at each touchpoint and tell their story.

#### Clinical experience

The KPC dedicated several meetings to mapping and analysing the ED pain management process, drawing on the collective clinical experience of the team. We used a visual map of the ED, which is organised into coloured zones, to systematically whiteboard pain management processes at each stage of the patient journey. A detailed summary of this mapping activity was circulated to all members of the KPC for confirmation. The mapping process facilitated clinician discussion and reflection on what mattered most in the transition through the department, generating more questions and assumptions around pain management at each stage of the journey. Each of these meetings were recorded and transcribed in conjunction with journal notes and photographs of group work.

Staff were invited to share their reflections and stories of pain management throughout our KPC meetings, building a deeper understanding of pain practices and generating questions for enquiry. Nurse-initiated analgesia (NIA) was a practice that prompted lots of discussion and raised numerous questions for the group. To further explore these questions the KPC reached out to influential nursing leaders (nurse manager, nurse educator, NPs) to deconstruct the clinical, political and physical processes surrounding NIA.

To build consensus around the evidence we had reviewed, the KPC completed a visioning activity to clarify our purpose in a way that could be communicated to the rest of the ED team. The use of a creative process to develop an initial vision created the space for deeper thinking and became a catalyst for discussion of what optimal pain care could look and feel like for our families and clinicians [16]. This activity also contributed to appraisal of the evidence as it represented the experience and values of the KPC.

#### Local evaluation

To investigate the local experience of pain management we undertook a retrospective audit of pain management indicators for all ED presentations in the previous year. A clinical audit protocol was developed by the research team to identify variables of interest including patient demographics, triage and medication records and pain indicators. To allow subgroup analysis of children with painful conditions, two experienced ED clinicians (SW and DH) independently and then by consensus identified potentially painful triage presentation categories and conditions. All data were extracted from the integrated electronic Medical Record (ieMR). We accessed the ED nurse educator database to audit the number of nurses approved to nurse-initiate analgesia. Analysis of local data added to our exploration of the context and provided the KPC with a baseline of pain management indicators.

### Synthesis of datasets and analysis

**Fig. 1:**
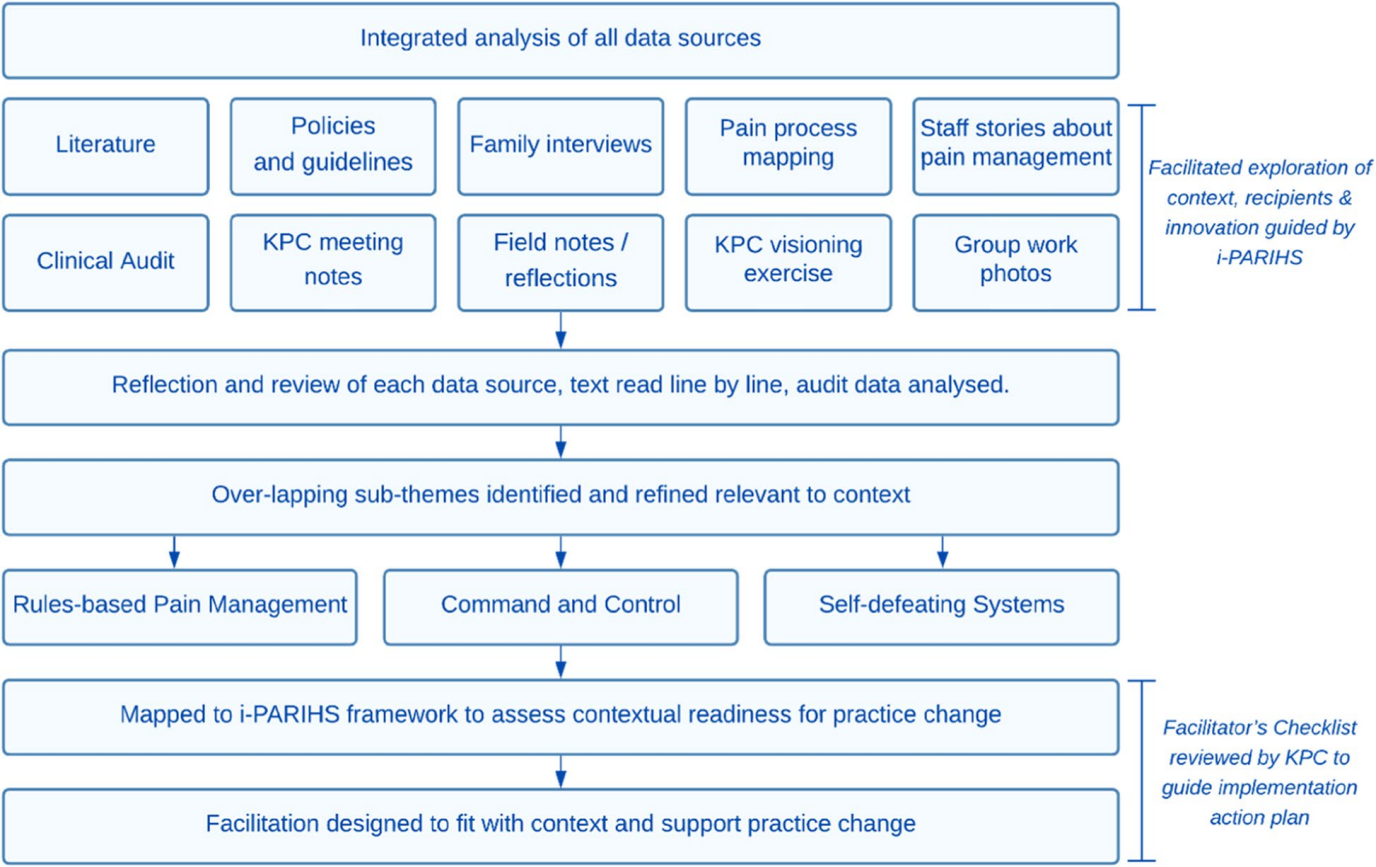
Data analysis and synthesis. *Note:* Adapted from Crowe and Manley [19]

Drawing on the principles of analysis described by Silverman [17], synthesis across datasets developed an account of the context and culture of pain care in the ED. Our process aimed to integrate all of the data inclusive of dimensions of evidence and context [6]. Each data source was individually reviewed through close reading of transcripts of interviews, meetings, journal notes, listening to audio recordings, and exploration of creative works. Audit data were summarised using simple descriptive statistics to explore patterns. We identified key ideas and concepts of interest, areas of overlap and noted interesting points. These concepts were coded, and similar ideas grouped under the same code. As new codes were developed, existing codes were reviewed and revised [17]. Through continued evaluation and analysis three primary overarching themes were developed: rules-based pain management, command and control, and self-defeating systems (Fig. 1). A selection of verbatim quotations has been included to illustrate these findings and “bring life to the text” from the perspective of the participating families and clinicians [18].

As we became immersed in these concepts, we drew on several theoretical frames to understand the prevailing culture and how pain management practices became established. In particular, given our focus on organisational change for optimising ED pain care [5], our process of evaluation and analysis was heavily influenced by systems thinking principles summarised in Table 1 [20]. Therefore, our goal was not to add more detail complexity by generating in-depth descriptive accounts of current practice, but instead to focus and plan collaborative implementation effort on those changes that might best optimise the whole system of pain care in the ED. The outcomes of this analysis provided structure and focus to inform our implementation plan.

**Table 1:** Systems thinking for change in complex systems ([20], p. 26)

• Motivates people to change because they discover their role in exacerbating the problems they want to solve	
• Catalyses collaboration because people learn how they collectively create the unsatisfying results they experience	
• Focuses people to work on a few key coordinated changes over time to achieve systemwide impacts that are significant and sustainable	
• Stimulates continuous learning which is an essential characteristic of any meaningful change in complex systems	

### Ethical approval

Ethical approval was obtained from the Children’s Health Queensland Human Research and Ethics Committee (HREC/19QCHQ/50738), and we received a Public Health Act approval (RD008011) to retrospectively access patient data from the hospital ieMR.

## Findings and discussion

### Establishing the kids pain collaborative: clarify and engage

Following the facilitator’s toolkit, we sought to engage clinicians and researchers around a shared interest in optimising ED pain care [6]. The toolkit provided a practical guide that we found useful given the size and complexity of the ED. We proposed a collaborative and participatory approach to evidence-based innovation in pain care, meeting with interdisciplinary ED leaders to establish an inclusive clinical-academic partnership. While ED leaders believed that pain management was done well in the department, they were open to reviewing current practice with the goal of optimising pain management at a department level.

With this endorsement we recruited staff to the KPC through a hospital-wide expression of interest which was disseminated through established ED communication channels by email, electronic bulletins and flyers. The PI attended ED handovers several times a week to initiate discussion about the project and encourage wide participation, and a SMO disseminated invitations at medical staff forums. The ED nurse unit manager further supported recruitment through email conversations, meetings and electronic announcements, offering nurses a range of options for attending the KPC meetings using dedicated education time or a reimbursement of time process.

Engagement with nursing and medical teams and inclusive recruitment at the beginning of the project engendered credibility and trust with clinicians. As a paediatric NP, the PI was well-known within the ED and wider hospital community, and able to draw on established partnerships as well as clinical experience to facilitate engagement. These connections proved to be invaluable in establishing and sustaining the KPC, especially in the context of facilitating discussions around policy and practice, as well as ongoing support and investment in the project.

### Understanding the context and culture of pain care: assess and measure

During the assess and measure phase, the KPC began meeting in March 2020 to explore the context, processes and culture of pain management within the ED. Our facilitation approach was pivotal to this process [11]. To promote engagement, we initially focused on team building, drawing on principles of i-PARIHS and Practice Development, using person-centredness to build relationships within the KPC and foster a shared exploration of pain management [6, 16, 21]. We identified and embedded agreed ways of working, developing an open respectful team relationship which was inclusive, supported listening and difficult discussions, and clarified expectations so that all participants could be heard, feel valued and speak freely. One external facilitator was an experienced Practice Development researcher and mentored the team in developing facilitation skills. During our first meeting we worked together to identify hopes, fears and expectations—establishing group rules, mutual respect and trust which grew as the meetings progressed [22].

Meeting on a fortnightly basis over 9 months, we continued to build the KPC using facilitation methods to set goals, build consensus and develop networks (inner and outer context) to support our reconnaissance of pain management evidence and context [6]. Following i-PARIHS, several weeks were spent collaboratively mapping and analysing ED pain practices and evidence from a range of sources [6]. Facilitation focused on supportive, curious questioning and active listening to enable KPC clinicians to explore practice based on their experience and knowledge [23]. This process provided the opportunity to unpack practice assumptions, to better understand what was working well and build consensus around how pain management could be optimised. Clinicians tended to jump to solutions early in the process of enquiry, proposing ideas around possible solutions which were held for future discussion when our contextual exploration was completed [16]. An important part of this journey was engagement with nursing leaders to undertake an in-depth investigation of NIA which evoked robust debate and triggered a review of the policy to achieve consensus and make recommendations for change. We also consulted the consumer representative group to clarify our understanding of pain management engagement and seek advice regarding communication between clinicians and families.

**Fig. 2:**
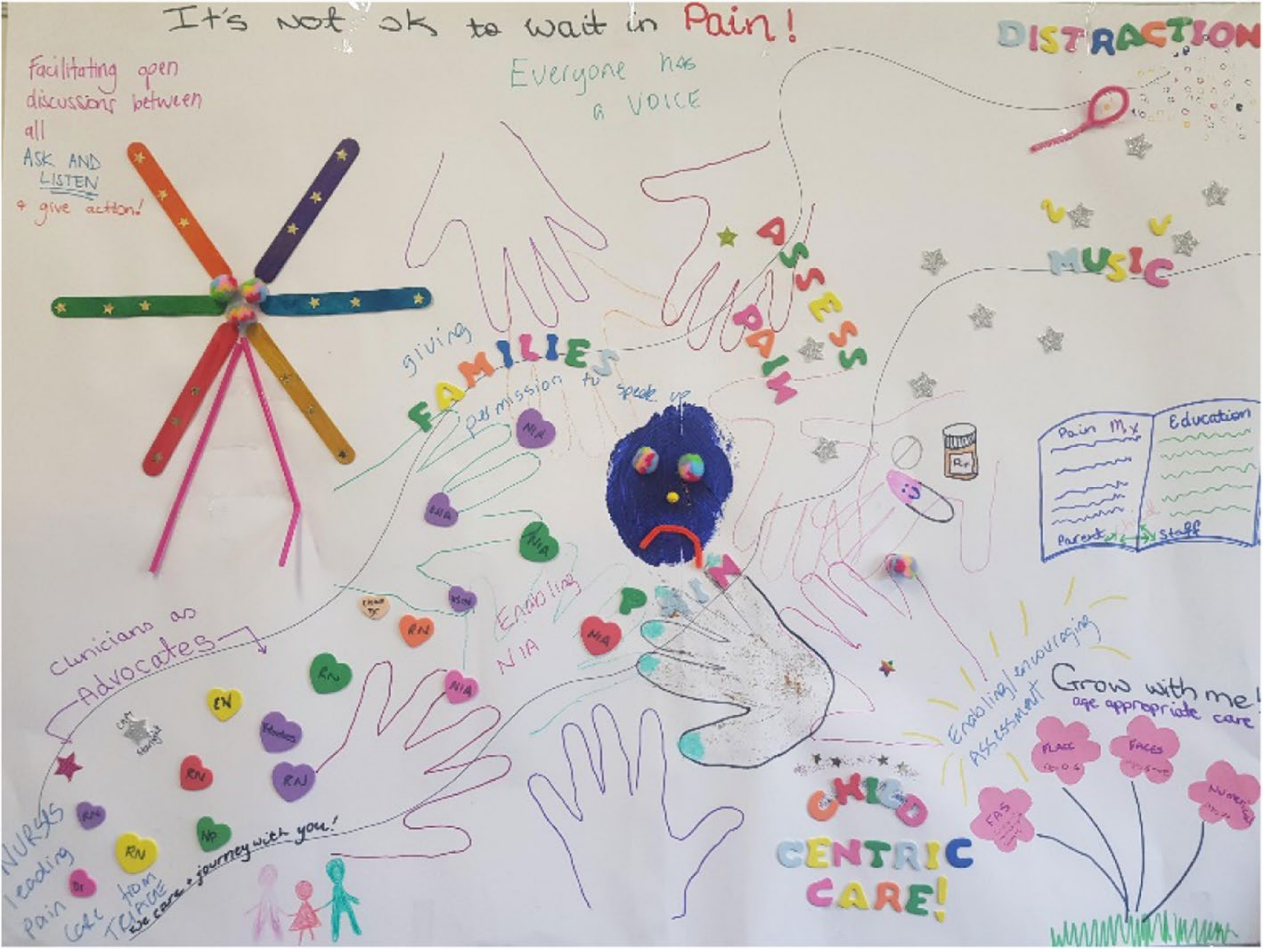
KPC visioning activity

Facilitation supported the group to clarify their purpose in ways that could be shared with the ED team to inspire and motivate values-based practice change. We engaged in a creative visioning activity [24], to create a space where we could reflect on what we had learnt, articulate our shared purpose and reach consensus around pain management solutions where families and clinicians all had a voice (see Fig. 2). This process led to the development of our KPC purpose statement: *It’s not ok to wait in pain.* To visually connect ED initiatives to the KPC, the Ferris Wheel drawn during the visioning activity became a symbol of the KPC facilitating clinicians to provide optimal pain care for children throughout the ED journey.

#### Boundary-spanning and building networks

To strengthen the KPC’s capacity to enable organisational change we sought stakeholder engagement with influential leaders in pain management [6]. The hospital Acute Pain Service nursing team became key members of the KPC, bringing extensive pain management knowledge and experience and the ability to influence the organisational context at the governance and policy level. These networks enabled the KPC to make evidence-based recommendations at the systems level to facilitate innovation.

#### The facilitator’s checklist

Towards the end of the assess and measure phase the KPC used the i-PARIHS Facilitator’s Checklist to crystalise an action plan [6]. This activity supported readiness for practice change, deepening our understanding of the innovation, recipients and context to inform a facilitation strategy for implementation.

This final step in evaluating the context, culture and leadership in ED gave the KPC confidence in the depth and completeness of our appraisal of contextual readiness. We structured an implementation plan shaped by these insights to reach out to as many ED staff as possible and facilitate a shared vision of pain care to inspire practice change. Resistance was expected from some clinicians, driven by the fear of increasing workload and the belief that current pain management processes were adequate. To overcome this barrier pain management additional change champions were recruited by targeting influential opinion leaders. Supported by a Queensland Government research grant, we were able to appoint two KPC members as part-time internal facilitators for the duration of the project to enable implementation with frontline staff.

### Unpacking assumptions: rules-based pain management

KPC meetings provided a safe, blame-free environment where staff could review their understanding of the complexities of pain management, bring together different perspectives and engage in the process of unpacking assumptions around practice. During our pain mapping exercise staff initially held strong views about how the pain management processes worked, but as we asked more questions it became apparent that team members each had a partial view of the pain management journey and some of their assumptions were contested by others. NIA became a focus of contradictions between policy, practice and culture which negatively impacted pain care.

Our appraisal of NIA was complex because of the extent of competing assumptions and rules governing practice which were deeply embedded in the ED culture and context. The group frequently revisited NIA as new practice assumptions would surface in meetings, such as the examples in Table 2.

**Table 2:** Example team assumptions and “rules” for nurse-initiated analgesia in the ED

• NIA must only be initiated at triage on the child’s arrival to ED	
• Analgesia (NIA or otherwise) could not be initiated at triage	
• Analgesia could not be initiated at triage if there is a queue	
• Analgesia could not be initiated if the ambulance service had given opioids prior to arrival	
• Only nurses who had attended a specific workshop could nurse initiate opioids	
• Only senior nurses could be approved to nurse initiate opioids	
• Policy required 3 or 4 registered nurses to initiate, check and administer analgesia	
• Enrolled nurses are not permitted to act as checkers for opioid medications	
• Approval to nurse initiate opioids was an individual practice choice	
• Analgesia could not be given or kept at triage	
• Nurses could not initiate analgesia if unable to weigh children: estimating weight is not permitted for analgesia but is permitted for other purposes	
• ED oral liquid opioid medication supply was limited to 20 ml bottles because of potential errors (measuring/administering)	
• Casual pool RNs (who worked regularly in the ED) were not approved to nurse initiate analgesia	
• NIA could not be initiated if the child had been allocated to a nurse practitioner or doctor	

Deconstructing the policy framework, associated rules (formal and informal) and cultural practices required many months of regular KPC meetings. This time was crucial to enable rigorous discussion and debate, ultimately achieving a comprehensive picture of how NIA was enacted from a systems and recipient level. When relevant the KPC also consulted with ED nursing and medical leaders to seek further clarification based on findings and secure support for proposed changes.

NIA had been introduced in the ED 5 years previously to improve access and timeliness of analgesia. The goal was to enhance nurse autonomy, as the first responders to pain at triage, to assess and initiate a first dose of analgesia within 30 min of the child’s arrival [5, 25]. The NIA policy framework was inclusive of simple analgesics (paracetamol, ibuprofen, topical anaesthetic gels) and opioids (intranasal fentanyl and oral oxycodone) so that nurses could independently manage mild, moderate and severe pain. The policy supported nurse-initiated opioids in the ED for nurse-led pain management throughout the episode of care from triage to discharge.

The KPC found, however, that the policy governing NIA, especially opioids, was rarely enacted. Approval to initiate non-opioid medications was integrated into a formal education pathway enabling nurses to initiate simple analgesia within 6 months of commencing in the ED. We identified that 70% of eligible nurses were able to nurse initiate simple analgesia, but the clinical audit revealed that only 33% of all analgesia administered to children was initiated by nurses (Table 3). This was much lower than expected given all children are initially assessed and triaged by a nurse. Approval to initiate opioid medications was not integrated at a systems level, but instead driven by individual choice. Uptake of this policy was very low with less than 10% of eligible registered nurses approved to initiate opioids; only 2.5% of opioids administered to all children and 3.1% to children with painful conditions were nurse initiated in the prior year (Table 3).

**Table 3:** Clinical audit of ED pain indicators

	All Children	Painful Conditions^**b**^
	***N =*** 72,735	***N =*** 28,615
**Pain score at triage by age**^**a**^	***n*** **= 32,042 (44.1%)**	***n*** **= 14,522 (50.8%)**
Newborn	628 (35.7)	42 (30.6)
Infant	7939 (39.9)	1594 (45.3)
Preschool	9141 (43.8)	3857 (49.1)
Child	10,038 (47.8)	6365 (52.4)
Adolescent	4296 (46.8)	2664 (53.6)
**Analgesia given by age**	***n*** **= 36,329 (49.9%)**	***n*** **= 18,628 (65.1%)**
Newborn	92 (5.2)	14 (10.2)
Infant	8685 (43.6)	1708 (48.6)
Preschool	10,545 (50.5)	4683 (59.6)
Child	12,164 (58.0)	8609 (70.9)
Adolescent	4843(51.7)	3614 (72.7)
**First analgesia administered**		
Fentanyl (intranasal)	1341 (3.7)	1002 (5.4)
Oxycodone (oral)	1289 (3.6)	956 (5.1)
Morphine (intravenous)	123 (0.3)	80 (0.4)
Paracetamol (oral, per rectum, intravenous)	19,304 (53.1)	8860 (47.6)
Ibuprofen (oral)	14,269 (39.3)	7730 (41.5)
**First analgesia initiated by**		
Medical Intern	545 (1.5)	161 (0.9)
Resident Medical Officer	6490 (17.9)	2208 (11.8)
Medical Registrar	12,110 (33.3)	5464 (29.3)
Senior Medical Officer	4032 (11.1)	1850 (9.9)
Nurse Practitioner	1080 (3.0)	849 (4.6)
Registered Nurse	12,071 (33.2)	8097 (43.5)
**First opioid analgesia initiated by**	***n =*** **6190 (%)**	***n =*** **4603 (%)**
Medical Intern	56 (0.9)	35 (0.8)
Resident Medical Officer	1157 (18.6)	793 (17.2)
Medical Registrar	3259 (52.7)	2442 (53.1)
Senior Medical Officer	1242 (20.1)	903 (19.6)
Registered Nurse	158 (2.6)	142 (3.1)
Nurse Practitioner	318 (5.1)	288 (6.2)
**Time to first analgesia by triage category**^**c**^	**Median minutes (IQR)**	**Median minutes (IQR)**
All categories	57.0 (31.0-104.0)	44.0 (25.0-81.0)
ATS1 (immediate)	37.0 (17.0-112.0)	26.0 (13.0-64.0)
ATS2 (10 min)	43.0 (23.0-106.0)	29.0 (18.0-50.0)
ATS3 (30 min)	59.0 (33.0-107.0)	45.0 (26.0-82.0)
ATS4 (60 min)	60.0 (32.0-103.0)	48.0 (27.0-86.0)
ATS5 (120 min)	56.0 (30.0-93.0)	46.0 (26.0-80.0)
**Time to first analgesia by age**		
Newborn	152.0 (75.0-284.0)	75.0 (50.0-85.0)
Infant	75.0 (40.0-124.0)	55.0 (31.0-98.0)
Preschool	61.0 (33.0-109.0)	48.0 (26.0-87.0)
Child	49.0 (28.0-91.0)	43.0 (25.0-79.0)
Adolescent	45.0 (24.0-84.0)	39.0 (23.0-73.0)
**Time to first opioid analgesia by age**		
Newborn	141.5 (80.5-208.0)	80.5 (59-102)
Infant	85.0 (40.0-152.0)	68.0 (32-131.0)
Preschool	86.0 (40.0-143.0)	80.0 (35.0-135.0)
Child	67.0 (32.0-129)	61.0 (29.0-124.0)
Adolescent	57.0 (29.0-114.0)	52.0 (28.0-106.0)
**Time to first analgesia initiated by**		
Medical Intern	97.0 (59.0-150.0)	78.0 (36.0-129.0)
Resident Medical Officer	90.0 (49.0-141.0)	72.0 (35.0-124.0)
Medical Registrar	72.0 (38.0-126.0)	56.0 (31.0-98.0)
Senior Medical Officer	63.0 (34.0-111.0)	49.0 (28.0-89.0)
Registered Nurse	38.0 (23.0-66.0)	34.0 (21.0-59.0)
Nurse Practitioner	53.0 (33.0-93.0)	50.0 (31.0-91.0)
**Time to first opioid analgesia initiated by**		
Medical Intern	102.0 (52.0-193.0)	88.0 (41.0-148.0)
Resident Medical Officer	83.0 (34.0-152.0)	73.5 (29.0-141)
Medical Registrar	76.0 (35.0-135.0)	66.0 (32.0-123.0)
Senior Medical Officer	59.0 (30.0-123.0)	52.0 (27.5-106.0)
Registered Nurse	26.5 (20.0-39.0)	25.0 (20.0-38.0)
Nurse Practitioner	90.5 (48.0-134.0)	85.0 (44.5-131.5)

^a^Age ranges [26]: Newborn: Birth to 1 month, Infant: > 1 month to < 24 months, Preschool: 2 years to < 6 years, Child: 6 years to < 13 years, Adolescent: 13 years to < 17 years

^b^Painful conditions by triage presentation categories [27]: abdo/pelvis/perineal pain; back pain; bite/sting; blunt injury; bruising/other; burn/scald; chest pain; crash other; cycle related; ear pain; electrocution; eye pain; face-other pain; fall; headache; laceration/skin tear; limb/joint pain; MBC/quad bike-driver; MBC/quadbike-passenger; multiple pain; MVC-driver; MVC- passenger; neck/throat pain; pedestrian vs; penetrating injury; strangulation/asphyxia; suspected foreign body/choking; swallowing difficulty; swelling/oedema/lump; unsettled

^c^Australasian Triage Scale [12]: categories and maximum waiting time for assessment and treatment

Uptake of the NIA opioid policy was not the only barrier. This practice was tightly governed by, albeit well intentioned, a formal rule because of perceived safety risks. Medications required double-checking during administration by nursing staff, but the policy mandated that the nurse initiating the opioid medication could not act as a checker during this process. The unintended consequence was that at least three (or more) registered nurses were required to nurse initiate opioids: one to prescribe, one to check and one to administer. This complex checking process was impractical and unachievable in a busy ED context. Yet it remained unchallenged, perpetuated by nurses themselves as a safety mechanism. Rather than questioning the policy, nurses requested opioid prescriptions from medical staff: “it’s quicker to ask a doctor to write up the fentanyl”. However, secondment of yet another clinician worked against analgesia timeliness and safety as the medical officer consulted was unfamiliar with the child in question, necessitating a secondary assessment prior to writing a prescription. In essence this rule delayed prescription and administration of analgesia to children, and deterred nurses from enacting a practice that is safe and effective [5]. Although it was designed to enhance nursing autonomy, the policy as implemented worked against nurses by restricting professional judgement and practice [28].

Implementing the NIA opioid policy was further confounded by assumptions that had emerged as informal rules and were enforced without question. Many “new rules” surfaced in KPC meetings over several months (e.g., Table 2). Nurses adhered to rigid, albeit unwritten rules around who could decide to give NIA, when, where, and under what circumstances. For example, many nurses believed they could not initiate analgesia for a patient in pain being treated by a doctor or NP, or who had been bought in by ambulance. Other nurses enforced that weight estimation was not permitted for NIA (in the case of injury it is not always possible to weigh the child). Given weight is required to calculate medication doses, standard paediatric practice is to estimate the child’s weight using a weight estimation tool. Instead of following standard practice, nurses would seek out a medical officer to estimate the child’s weight and write a prescription. The medical officer often had limited paediatric experience in comparison to the paediatric nursing team who routinely estimated weights of children when providing emergent care. The KPC unpacked many other assumptions which impeded NIA. These assumptions around NIA were contested—audit data clearly demonstrated that even with only 33% (all children) and 43% (painful conditions) of all analgesia being nurse initiated, this practice reduced median time to analgesia for all children from 57 to 38 min and for children with painful conditions to 34 min. However, we found these assumptions were accepted by the majority and reinforced by irrational practices, working against pain management. There was no shared understanding of this practice and the potential to improve pain management. When NIA was discussed at a meeting of more than 20 SMOs, it was clear that the majority were not aware of the nurse-initiated opioid policy, or the need to support nurses in enacting this policy to optimise timely analgesia. There was also a distinct lack of confidence in nurse-initiated opioids, evidenced by the voiced concern of some SMOs and refusal of some nurses to administer opioid analgesia initiated by their nursing colleagues. Discussion centred on avoiding safety risks rather than questioning these assumptions and the policy framework.

Unwritten rules, created by nurses, controlled nursing practice and contributed to the dysfunction of policy intended to facilitate analgesia. Rather than circumventing children waiting in pain, the policy and practice assumptions created even longer delays in ways that removed the child and family even further from the centre of care. Nurses enforced these assumptions, which were representative of individual beliefs and opinions rather than evidence, limiting the uptake and implementation of NIA. Nurses were complicit in constructing their practice in a way where accountability was to follow the rules and assumptions rather than to the families and patients, especially at triage. The system sustained this unquestioning reliance on rules rewarding adherence irrespective of consequences to children and their families, because following the rules was valued:

*Compliance to giving medication to children with arm bands (identification band) on is rewarded, if we give analgesia to a child without an arm band, we get picked up on it. But sometimes the admin staff can take a long time to put on the armband on if they are busy, which means the child has to wait longer for analgesia.* (RN)

Pain management assumptions were embedded across all levels of the system, accepted without question as the cultural norm as a way of mitigating risk, and nurses without authority did not have the power or confidence to question policy or rules. NIA practice was constructed around rules which took precedence over judgement, contributing to system complexity while eroding the nurse’s professional capability and standing within the team. Bail et al., refers to this as a “theory-practice contradiction” where procedural policy, under the auspices of patient safety, overrides autonomous, critical thinking [28]. The consequences of these rules, which undermine reflective practice, were breakdowns in pain management at the point of care.

While the KPC and broader nursing team articulated a values-based model of practice that placed the child and family in the centre, rules-based pain management worked against this. This disconnect between values and practice was the product of a workplace culture where nurses were not encouraged to challenge or question their practice, accepting taken-for-granted practices rather than evaluating their effectiveness [29].

### Command and control

In this high-acuity setting the pervasive focus was, understandably, prevention of mortality and morbidity and getting the job done—and rules and policies such as NIA were in place to ensure standardised practice to protect children and families. With more than 75,000 presentations a year and a staff base exceeding 250 clinicians, a top-down hierarchical model of nursing and medical leadership had been adopted to support this high volume of patients, minimise risk and increase efficiency. ED members of the KPC described a culture of command and control, with an emphasis on task orientated care provision as a way of managing and organising complexity in an environment where a critically ill child could arrive unannounced at any moment. This cultural imperative drove the unquestioning acceptance of rules-based pain management by nurses, exemplified by NIA [30, 31]. The transactional nature of organising practice relied heavily on policies and rules to create order and clarify expectations, rewarding compliance and productivity at the department level. For example, patient flow was focused on meeting the National Emergency Access Target (known as the 4 h rule) of discharging patients with 4 h of presentation and compliance to the Australian Triage Scale waiting times [12, 32] was closely monitored and reported as a quality indicator.

As a consequence of top-down leadership, nurses who were not in a designated management role were not trusted to make independent decisions unless they had seniority by length of service. Permission to make decisions and act was regulated by these formal leaders in a system established to follow direction and focus on tasks. Although well intentioned, this control implied a fundamental mistrust of nurses impeding their ability to act or make decisions independently. Nurses who were not in leadership roles tended to accept the status quo unquestioningly, as we have seen in NIA.

Another example was the departmental rule of triaging patients within 3 min. This task orientated approach to care had unintended consequences that worked against quality pain care. Rapid triage undermined assessment and treatment of pain at a key point in the ED journey—for pain to be well managed it must be recognised and acted upon at triage [5, 12]. Nurses were torn between policy and following the rules, stifling their ability to think critically and provide person-centred care:

*NIA at triage is problematic from this perspective as it adds to the triage time. RNs feel pain can’t always be prioritised depending on what was wrong with the child and other factors such as line up at triage. They feel pressured to triage quickly by parents waiting in the queue and policy.* (RN)

With competing organisational demands, the urgency of pain care could easily be overlooked. Local data showed only 44% of children had documented pain scores at triage and the median time to analgesia for all presentations was 57 min and 44 min among painful conditions (Table 3).

The focus on rapid triage also proved to be a barrier to communication with families and children around pain, as it minimised the interaction between the triage nurse and family. The transactional nature of pain management on arrival at triage was reported by parents who were waiting for direction, felt disempowered in an unfamiliar environment and were seeking guidance and permission to speak about pain:

*At the [triage] desk it was more of a transactional discussion. The nurse didn’t ask anything much about Max’s pain. He didn’t get anything for pain even though I thought it was severe at the time. I told her he was in bad pain – she didn’t ask anything about numbers or really talk to Max directly. We didn’t really know if we could ask for pain relief, and it wasn’t offered. We felt unsure and uncertain, and we were waiting for that leadership.* (Parent)

It was evident that the conflicting demands of the 3 min triage and need to maintain flow and undermined the nurse’s ability to connect with parents or prioritise pain management. Families felt overwhelmed in the ED environment and disconnected from nurses. They were unsure of how to behave, what was expected of them, or who to talk to; they waited for nurses to give them direction and ask them about their child’s pain. Several parents reported that nurses seemed powerless to help with pain perceiving pain management to be the job of medical officers.

Task orientated triage negated the ability for nurses to be person-centred in the triage process or establish a therapeutic relationship with parents, in contrast to what families want: emotional support, communication and timely alleviation of their child’s pain [5, 33].

*It’s a bit like getting on a train – you just feel like the nurses and doctors are in control and know what they are doing, so you just sit there. I didn’t know what to ask for and sort of assumed if they could do anything else they would have. We were on the train!* (Parent)

It is not surprising, given the social and political agendas around the pressures on access to ED care, that we see great emphasis on efficiency and time-to-task outcomes that filter down in organisations to ED managers and staff. Within this context principles of scientific management have infiltrated organisational life in seeking to achieve the most productive and efficient performance. We value what we can measure (time to analgesia, KPIs), rather than measuring what we value (safe, effective, person-centred care).

In responding to the complexity inherent in ED work, efforts are directed to reducing risk to as low as acceptable by overcoming potential for mistakes through competency training, protocols and procedural policies detailing how “Work-As-Imagined” (by those distanced in time and space from the frontline) must be performed [34]. Ensuring compliance between Work-As-Imagined and Work-As-Done in the messy and complex reality of ED nursing is established and enforced in the workplace culture as “rules”. Rules emerge as both formal and informal rules. And while “rules are primarily the creations of actors, once established, they appear as structures standing over and above people” ([34], p. 56).

The power of “rules” for pain management is both structurally reproduced through a command-and-control model of leadership – and also enacted through a lens of disciplinary power by nurses holding themselves and each other to account for misunderstanding, resisting or breaking the rules. Nurses consent to “writing themselves into a web of obedience” in policy and practice [28]. Rules can make irrational and self-defeating practices seem like a plausible option for reducing risk (e.g., removing simple analgesia from unlocked draws in triage as nurses cannot be trusted; withholding analgesia without weighing scales).

Whereas rule-following promotes standardisation and linear thinking, the complexity of ED practice requires variation and adjustments responsive to the demands of the context and situation – which is necessary for safe and effective performance (as Hollnagel argues in the concept of Safety-II) [34]. Pain management “by the rules” is organised in highly hierarchical ways, and as required by competing organisational demands, can be delayed or withheld.

Leadership seeking to democratise power draws on the skills and expertise of the collective [35]. A model of distributive rather than transactional leadership, facilitating clinician autonomy, has the potential to empower authentic engagement and partnership around pain care between families and clinicians [36]. We have glimpsed this idea of distributive leadership in some of the reflections shared within the KPC, where nurses strive to support pain management by engaging with other nurses to facilitate best practice around NIA and triage. Nurses have the potential to lead transformational workplace culture given they are the largest professional group in the ED team and spend more time with patients and families than any other discipline [36]. There is an opportunity to build team capacity to optimise evidence-based pain management through trust and respect, valuing and promoting autonomy, and collective leadership [37].

### Self-defeating systems

As a consequence of rules-based pain management and the command and control approach to leadership, self-defeating practices had emerged that worked against person-centred pain care and disconnected the team. The focus on management of emergent care and patient flow had become self-defeating for pain care. Ways of working as a team were not embedded at the systems level—nurses and doctors were observed to work alongside each other in parallel: nurses focusing on tasks, doctors focusing on seeing and discharging patients. The imperative of avoiding safety risks and maximising patient flow through the ED had created a fragmented approach to care and creation of systems which, in striving to manage complexity, undermined pain care.

Fragmentation was evident in the absence of continuity of pain care. When families were moved around the ED as part of the flow management strategy, critical conversations about pain care or handover of a pain care plan were uncommon. Children moving between ED zones or waiting for admission by the inpatient team did not routinely have analgesia prescribed by their treating clinician. The rules surrounding NIA stopped nurses working proactively and autonomously. Children waited in pain because, as reported in the literature, pain scoring itself often did not trigger appropriate intervention [5, 38]. Connected, joined-up teams in the ED are essential for effective pain care.

Because ED systems of care are focused on patient flow and efficiency above all, these assumptions were not challenged at the team level, despite the impact on quality of care. For example, the team had created a clinical initiatives nurse (CIN) role to, as its first priority, assess and monitor children’s pain in the waiting room after triage. Yet nurses working as the CIN were routinely seconded by SMOs to assist with procedures in other areas of ED, irrespective of the pain management needs of the children in the waiting room, to optimise patient flow:

*I know we need to give the kids in the waiting room pain management but that’s not our priority. My CIN can’t be out in the waiting room giving out pain relief I need her inside helping me with procedures to keep the flow going.* (SMO)

Nurses understood the negative impact of the CIN secondment on pain care, reporting that failure to relieve pain increased length of stay for many children because pain prevented assessment and investigations such as x-ray. Yet reallocation of the role was rarely challenged. Nurses felt powerless, irrespective of the number or needs of children waiting in pain, acquiescing to direction by the medical team, sacrificing quality of care to patient flow. The self-defeating practice, designed to improve efficiency and patient flow, discounted the complexity of the system as a whole, and ultimately contributed to pain and distress, working against ED flow and undermining nursing autonomy [20].

Pain care was also impacted by the transient population of training medical officers because of inexperience, lack of knowledge around pain management processes and reluctance to listen to nurses:

*New doctors are afraid of giving opioids to children and they don’t listen to our advice at the start.* (RN)

New medical officers lacked confidence in prescribing opioid analgesia for children in moderate or severe pain, but were distrustful of nursing knowledge and experience, countermanding NIA and refusing to prescribe opioids themselves. Nurses were forced to seek ad hoc support from SMOs in these situations, which further delayed analgesia for children. Because there was little opportunity to share and reflect as an interprofessional team on pain care or engage in experiential team learning on the effectiveness of the current practice, breakdowns in care were expected with each new cycle of medical officers.

The facilitated process of critical reflection with the KPC, although at times uncomfortable, raised awareness that staff were reproducing systems of care, even organisational practices designed to optimise pain outcomes (e.g., NIA policy and CIN role), in ways that worked against what they wanted to achieve as a team. This insight was a powerful catalyst in motivating change [20, 24]. As others have found [39] we noticed that the process of collective enquiry itself was enough for KPC members to initiate several practice changes before any implementation action cycles began in the ED. Our reconnaissance gave the KPC foci for action to optimise pain care as a collaborative and facilitate a culture where pain management is a priority. Based on this work the next phase of the project is focused on: prioritising pain management and enabling NIA, promoting family involvement, and embedding team reflection, evaluation and learning for effective pain care.

## Conclusion

Reflecting on the value of the methodology and the implications for future research, our project underscores the need for, and value of, investing in authentic stakeholder engagement, collaborative appraisal of context and co-creation of innovation using skilled facilitation. We have presented an example of prospectively applying i-PARIHS with frontline ED staff to generate context-specific knowledge to inform facilitation and implementation. Using a systems thinking approach clinicians were able to apply a critical lens to their practice to better understand how pain management could be improved by letting go of the cultural practices they had created that worked against effectiveness and communication with families [20]. An embedded researcher model also facilitated clinician engagement and sustainability of future practice change [10].

Developing authentic and respectful relationships was integral to this first step in redesigning pain management to reach for systems level solutions [16]. Without clinician engagement and commitment to reflect deeply and honestly on their practice, a comprehensive understanding of the context of pain management would not have been possible. Facilitation is a far more powerful intervention than education and was the key ingredient to working through this complex process of building consensus on current practice to inform innovation. We successfully facilitated this engagement through intentionally person-centred ways of working, which were participatory and collaborative, enabling us to develop a shared purpose which placed the child and family at the heart of the project.

Our goal in establishing the KPC partnership was to facilitate effective workplace culture where staff are collectively focused on person-centred pain care and can critically evaluate practice rather than following policy without questions. Skilled facilitation in the setting of an authentic partnership has the potential to close the gap between evidence, vision and practice by enabling a bottom-up approach to reflection on, and redesign of, clinical practice. By challenging and questioning our practice in this way we, as clinicians and researchers, can learn to evaluate the effectiveness of what we do together and surface assumptions about what constitutes good practice.

Moving forward the KPC will focus on a small number of significant systems level changes with the ED team to achieve a cultural shift and support sustainability in pain management practice. This shared approach to assessing contextual readiness for optimising practice can be applied to any clinical problem through enabling clinicians, researchers and patients to have a voice in how we do things.

This research provides an example of a theoretically-driven and collaborative approach to implementation underpinned by i-PARIHS. Research collaboration to influence workplace culture and inform ED practice change requires respectful and authentic partnerships between clinicians and researchers. Using the principles of i-PARIHS we were able to engage key stakeholders and facilitate a process of collaborative enquiry where clinicians and researchers could openly and honestly evaluate the context and culture of pain management. The KPC is now facilitating implementation action cycles to create new embedded ways of working: a workplace culture where it’s not ok to wait in pain.

## Supplementary Information

**Additional file 1.** Children characteristics included in the pain audit.

## Data Availability

The datasets analysed during the current study are not publicly available due to the anonymity requirements of the ethic permissions granted but are available from the corresponding author on reasonable request and with the permission of the funding body.

## Abbreviations

CIN: Clinical Initiatives Nurse
ED: Emergency Department
i-PARIHS: Integrated Promoting Action on Research Implementation in Health Services
KPC: Kids Pain Collaborative
NIA: Nurse Initiated Analgesia
RN: Registered Nurse
SMO: Senior Medical Officer
