# Supplementary material for: Building effective engagement for implementation with i-PARIHS: a collaborative enquiry into paediatric pain care in the emergency department
Source: BMC Health Serv Res. 2022 Mar 12;22:330. doi: 10.1186/s12913-022-07740-w (PMC8916941; doi:10.1186/s12913-022-07740-w)
Supplement: Supplementary file 1 — Additional file 1. Children characteristics included in the pain audit. [file 12913_2022_7740_MOESM1_ESM.docx]

**Supplementary File: Children characteristics included in the pain audit**

|  | **All Children** |  |
| --- | --- | --- |
| **Characteristics** | ***N* = 72735** | **%** |
| **Gender** | | |
| Male | 40401 | 55.5 |
| Female | 32331 | 44.5 |
| **Age^1^** | | |
| Newborn | 1758 | 2.4 |
| Infant | 19910 | 27.4 |
| Preschool | 20882 | 28.7 |
| Child | 20994 | 28.9 |
| Adolescent | 9191 | 12.6 |
| **Australasian Triage Score (ATS)** | | |
| ATS 1 | 380 | 0.5 |
| ATS 2 | 10842 | 14.9 |
| ATS 3 | 24715 | 34.0 |
| ATS 4 | 33389 | 45.9 |
| ATS 5 | 3409 | 4.7 |
| **Presentations by Days of Week** | | |
| Monday | 10912 | 15.0 |
| Tuesday | 10329 | 14.2 |
| Wednesday | 10048 | 13.8 |
| Thursday | 9673 | 13.3 |
| Friday | 10072 | 13.9 |
| Saturday | 10444 | 14.4 |
| Sunday | 11257 | 15.4 |
| **Referral to ED** | | |
| Self | 57340 | 78.8 |
| General Practitioner | 8836 | 12.2 |
| Other Hospital | 4144 | 5.7 |
| Police | 591 | 0.8 |
| Other | 508 | 0.7 |
| Outpatients Clinic | 424 | 0.6 |
| 13-Health (telehealth) | 423 | 0.6 |
| Community Services | 310 | 0.4 |
| Specialist | 159 | 0.2 |
| **Mode of Arrival** | | |
| Self | 59085 | 81.2 |
| Ambulance | 13242 | 18.2 |
| Police | 295 | 0.4 |
| Other | 113 | 0.2 |
| **Discharge Destination** | | |
| Home | 53749 | 73.9 |
| Short Stay Unit | 8937 | 12.2 |
| Inpatient ward | 8048 | 11.0 |
| Did not wait | 1291 | 1.7 |
| Other hospital | 325 | 0.4 |
| Operating Theatre | 140 | 0.1 |
| Other | 137 | 0.5 |
| Outpatients | 42 | 0.1 |
| Hospital in the Home | 28 | 0.0 |

*Note:* ^1^Age ranges (42): Newborn: Birth to 1 month, Infant: >1 month to < 24 months, Preschool: 2 years to < 6 years, Child: 6 years to < 13 years, Adolescent: 13 years to < 17 years
